# Critical role for *CaFEN1* and *CaFEN12* of *Candida albicans* in cell wall integrity and biofilm formation

**DOI:** 10.1038/srep40281

**Published:** 2017-01-12

**Authors:** Md. Alfatah, Vinay K. Bari, Anubhav S. Nahar, Swati Bijlani, K. Ganesan

**Affiliations:** 1 CSIR-Institute of Microbial Technology, Sector 39-A, Chandigarh-160 036, India

## Abstract

Sphingolipids are involved in several cellular functions, including maintenance of cell wall integrity. To gain insight into the role of individual genes of sphingolipid biosynthetic pathway, we have screened *Saccharomyces cerevisiae* strains deleted in these genes for sensitivity to cell wall perturbing agents calcofluor white and congo red. Only deletants of *FEN1* and *SUR4* genes were found to be sensitive to both these agents. *Candida albicans* strains deleted in their orthologs, *CaFEN1* and *CaFEN12,* respectively, also showed comparable phenotypes, and a strain deleted for both these genes was extremely sensitive to cell wall perturbing agents. Deletion of these genes was reported earlier to sensitise cells to amphotericin B (AmB), which is a polyene drug that kills the cells mainly by binding and sequestering ergosterol from the plasma membrane. Here we show that their AmB sensitivity is likely due to their cell wall defect. Further, we show that double deletant of *C. albicans* is defective in hyphae formation as well as biofilm development. Together this study reveals that deletion of *FEN1* and *SUR4* orthologs of *C. albicans* leads to impaired cell wall integrity and biofilm formation, which in turn sensitise cells to AmB.

*Candida albicans*, an opportunistic pathogen of humans, can cause infections ranging from superficial skin infections to life-threatening invasive infections^1^,^2^. Mortality due to invasive infections can be as high as 75% worldwide^3^,^4^. Though several antifungals are available for treatment, they suffer from lack of broad spectrum of activity, drug resistance or high cost^4^. Since fungi are eukaryotes they share a large number of cell processes with their mammalian hosts and thus the number of drug targets are limited^5^. However, ergosterol and cell wall that are present in fungi but absent in their hosts serve as targets of commonly used antifungals^5^,^6^. While antifungals such as azoles and terbinafine inhibit ergosterol biosynthesis, amphotericin B, a polyene antifungal, binds to ergosterol of plasma membrane and kills the cells primarily by sequestering ergosterol^5^,^7^,^8^. Among antifungals that target cell wall, echinocandins and nikkomycin inhibit β-Glucan synthesis and chitin synthesis, respectively. Defective cell wall biogenesis is known to attenuate infections by *C. albicans*^9^,^10^. Thus, a better understanding of cell wall biogenesis and integrity may reveal novel targets for antifungal drug development.

Yeast cell wall is a complex network of polysaccharides (β-1,3-glucan, β-1,6-glucan and chitin) and mannoproteins^11^,^12^. β-1,3-glucan and mannoproteins are major components of cell wall comprising 30–45% and 30–50%, respectively. β-1,6-glucan and chitin contribute to 5–10% and 1.5–6.0% of the total cell wall biomass^11^. The cell wall is involved in several protective functions of the cells, including stabilisation of intracellular osmotic balance, oxidative stress, heat stress and antifungal resistance^11^,^12^,^13^,^14^,^15^. Cell wall integrity (CWI) is maintained by PKC1–MPK1 (Slt2) pathway, which helps in the biogenesis of cell wall^16^,^17^,^18^,^19^. Sphingolipid pathway intermediates, such as dihydrosphingosine and phytosphingosine, are also involved in CWI signalling^20^,^21^. Sphingolipids are a class of sphingoid backbone or long chain base (LCB) containing lipids^21^,^22^. These are major components of eukaryotic membranes and are abundant in the plasma membrane. In *S. cerevisiae*, these constitute 30% of the total phospholipids and about 7% of the total mass of the plasma membrane^23^. Sphingolipids coexist with sterols and glycerophospholipids and define the integrity as well as the plasticity of plasma membranes^24^,^25^. In addition to providing a structural framework, along with sterols they form functional microdomains in plasma membrane called lipid rafts^26^,^27^, which are involved in many physiological activities of the cells, including actin organisation, endocytosis, signal transduction and cell morphogenesis^28^,^29^,^30^,^31^. Moreover, lipid rafts participate in the sorting of glycosylphosphatidylinositol (GPI)-anchor proteins to the cell surface^32^,^33^,^34^. About one-third of the identified GPI-anchor proteins of *S. cerevisiae* contributes to cell wall biogenesis and their deficiency results in cell wall defect^12^.

Though previous studies have shown the involvement of sphingolipids in the CWI signalling^20^,^21^, there are no reports regarding the role of individual sphingolipid biosynthetic pathway genes in CWI modulation. To identify these genes, first, we have screened deletion mutants of sphingolipid biosynthetic pathway genes of *S. cerevisiae* with cell wall perturbing agents calcofluor white (CFW) and congo red (CR) and found that only deletants of *FEN1* and *SUR4* genes were sensitive to both the chemicals. These strains also showed other phenotypes typical of mutants with impaired CWI. Next, we have checked if the orthologs of these genes in *C. albicans* have a similar role, and found that their mutants also have comparable phenotypes. Moreover, *C. albicans* strain deleted in both these genes was found to be defective in hyphae formation and biofilm development. Since these mutants are also amphotericin B (AmB) sensitive^35^, we have tested the correlation between these phenotypes and find that their AmB sensitivity is likely due to their impaired cell wall.

## Results and Discussion

### Deletants of *FEN1* and *SUR4* genes of *S. cerevisiae* and their orthologs in *C. albicans* are impaired in cell wall integrity

**Figure 1:**
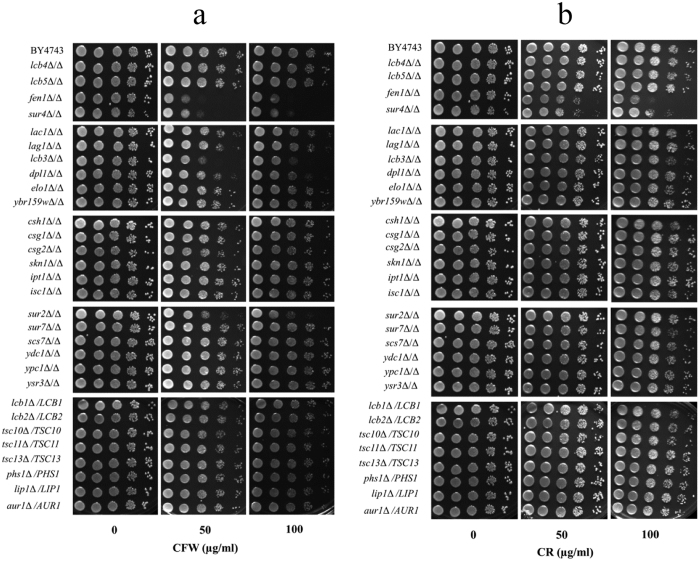
Screening *S. cerevisiae* deletants of sphingolipid biosynthetic pathway genes. An overview of sphingolipid biosynthetic pathway is shown in Fig. S1. Ten-fold serial dilutions of cells were spotted onto synthetic complete agar plates with indicated concentration of (**a**) calcofluor white (CFW), or (**b**) congo red (CR). Plates were incubated at 30 °C for 2 days before being photographed.

Though the role of sphingolipids in CWI signalling is known^20^,^21^, that of individual sphingolipid biosynthetic pathway genes is not yet reported. To identify such genes, we have screened homozygous deletants of twenty-two non-essential genes of the sphingolipid pathway of *S. cerevisiae* (Fig. S1), which were constructed as part of the yeast deletion project^36^, at various concentrations of CFW and CR. Cells with defective cell wall are known to increase chitin synthesis as a compensatory mechanism to maintain the cell wall integrity^11^,^12^. CFW and CR, which bind chitin, have been extensively used to identify such mutants since they are sensitive to a lower concentration of these compounds compared to normal cells^37^,^38^. Two deletants, *fen1*Δ/Δ and *sur4*Δ/Δ, were found to be sensitive to both the chemicals compared to the parent strain (BY4743) (Fig. 1). CFW sensitivity of *FEN1* deletant was also reported earlier, after being identified through a screen for genetic interaction with *CCW12*, a gene involved in CWI^39^. Among other deletants, *lcb3*Δ/Δ and *sur2*Δ/Δ were found to be slightly sensitive to CFW (Fig. 1a), but their growth on CR was comparable to the parent strain (Fig. 1b). While we cannot rule out moderate cell wall defect in these mutants, we initially focused our study on *FEN1* and *SUR4* deletants, since only these were sensitive to both CFW and CR, and thus definitely impaired in cell wall integrity. Heterozygous deletants of essential genes of sphingolipid biosynthetic pathway were also screened with the notion that if they are haploinsufficient, then their CFW and CR sensitivity can be determined. However, their growth was comparable to the parent strain (Fig. 1). *FEN1* (*ELO2*) and *SUR4* (*ELO3*) along with *ELO1* encode fatty acid elongases, which synthesise long chain or very long-chain fatty acids (LCFA or VLCFA)^21^,^40^,^41^. Elo1p, Elo2p (Fen1p) and Elo3p (Sur4p) are involved in the synthesis of C14 to C16 LCFA, up to C24 VLCFA, and C24 or C26 VLCFA respectively^21^,^40^,^41^,^42^ and mutations in *FEN1* and *SUR4* genes result in shortened fatty acid chains and lower levels of sphingolipids^40^,^42^.

**Figure 2:**
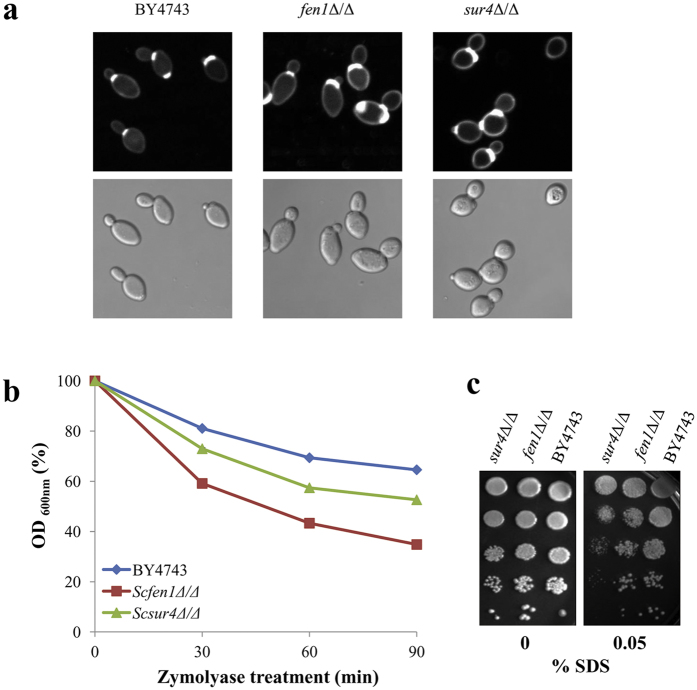
*FEN1* and *SUR4* deletants of *S. cerevisiae* are impaired in cell wall integrity. (**a**) Deletion of *FEN1* and *SUR4* genes increases chitin content in the cell wall. Cells of the parent (BY4743) and deletants were stained with CFW, and images were captured at identical conditions with a fluorescence microscope using 100× objective lens (upper panel). DIC images of corresponding fields are shown in the lower panel. (**b**) *fen1*Δ/Δ and *sur4*Δ/Δ strains are more sensitive to zymolyase. Zymolyase digestion of the parent and deletants was monitored by periodically measuring OD_600nm_ until 90 minutes. Average values of two independent experiments, carried out in triplicate each time, are shown. (**c**) SDS sensitivity of *fen1*Δ/Δ and *sur4*Δ/Δ strains. Cells were spotted onto synthetic complete agar plates with indicated concentration of SDS and incubated at 30 °C for 2 days before being photographed.

Since cell wall mutants that are sensitive to CFW and CR often have more chitin in their cell wall^11^,^12^,^37^, we used CFW staining to visualise chitin. More intense and larger area of fluorescence was seen at bud scars and mother-daughter cell junctions of *Scfen1*Δ/Δ and *Scsur4*Δ/Δ strains. Semi-quantification of fluorescence intensity using the NIS-Elements software showed that *Scfen1*Δ/Δ and *Scsur4*Δ/Δ strains have 27% and 32%, respectively, higher intensity than the parent strain, confirming that these mutants indeed have more chitin (Fig. 2a). Since chitin level is also increased in mutants impaired in the synthesis of β-1,3-glucan^12^,^43^, we speculated that *Scfen1*Δ/Δ and *Scsur4*Δ/Δ strains may be defective in β-1,3-glucan synthesis. Cells with decreased levels of ß-1,3-glucan, or increased accessibility of β-1,3-glucan due to some other defect in the cell wall, are more sensitive to zymolyase having β-1,3-glucanase as its principal constituent^44^. *Scsur4*Δ/Δ strain was found to be sensitive to zymolyase compared to the parent strain and *Scfen1*Δ/Δ strain was even more sensitive (Fig. 2b), which is comparable to their relative sensitivity to CFW and CR (Fig. 1). Next, we tested the sensitivity of these deletants to SDS, a detergent usually employed to determine the compactness of the cell wall, since less compact cell wall would allow SDS to readily reach and damage the plasma membrane resulting in cell death^9^,^45^. *Scsur4*Δ/Δ strain was found to be SDS-sensitive (Fig. 2c) suggesting that it has a less compact cell wall structure; however, altered lipid composition of the plasma membrane might have also rendered it more susceptible to SDS.

**Figure 3:**
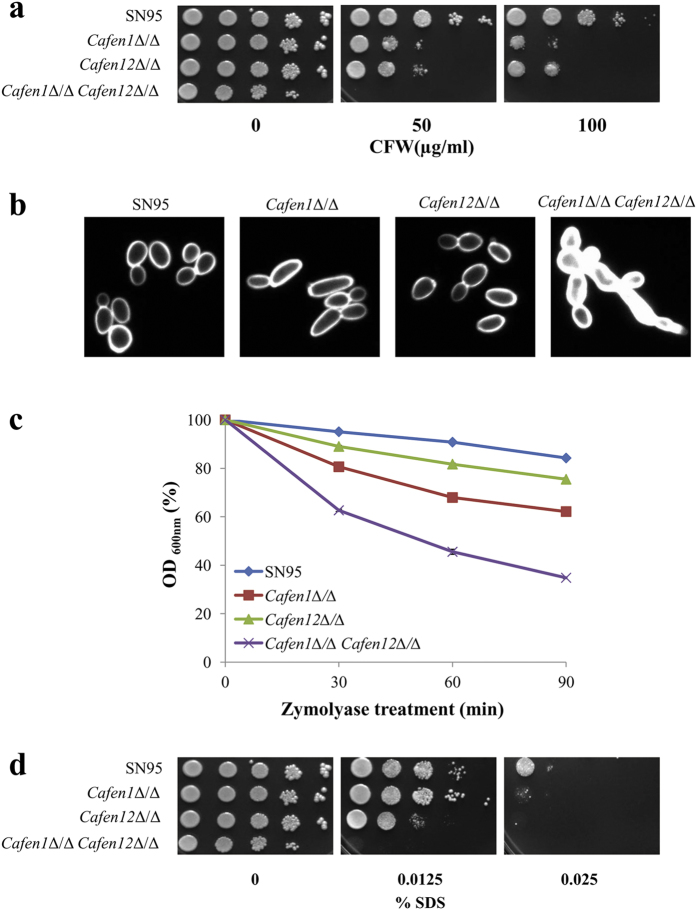
*CaFEN1* and *CaFEN12* deletants of *C. albicans* are impaired in cell wall integrity. (**a**) *C. albicans* strains deleted in *CaFEN1* and *CaFEN12* genes are hypersensitive to CFW. Cells of the parent (SN95) and single as well as double deletant strains of *CaFEN1* and *CaFEN12* genes were spotted onto synthetic complete agar plates with indicated concentration CFW and incubated at 30 °C for 2 days. (**b**) Chitin level of single and double deletants of *CaFEN1* and *CaFEN12* genes. After CFW staining images were captured under identical conditions with a fluorescence microscope using 100× objective lens. (**c**) Zymolyase sensitivity, determined as described in Fig. 2b. (**d**) SDS sensitivity. Cells of the parent and deletants were incubated with indicated concentration of SDS as mentioned in Fig. 2c.

*CaFEN1* and *CaFEN12* are orthologs of *ScFEN1* and *ScSUR4*, respectively, in *C. albicans*^35^. To check if these are also involved in cell wall integrity, we tested the sensitivity of their deletion mutants to cell wall perturbing agents. Similar to the deletants of *ScFEN1* and *ScSUR4*, deletants of *CaFEN1* and *CaFEN12* showed comparable sensitivity to CFW. Moreover, the strain deleted in both *CaFEN1* and *CaFEN12* was found to be hypersensitive to CFW (Fig. 3a). To check that the phenotypes seen with the double delete strain are actually due to deletion of these genes, and not because of any extraneous mutation, reintegrant strains were constructed by introducing wild-type *CaFEN1* or *CaFEN12* genes at their respective loci in the double delete strain. This has resulted in the suppression of the sensitivity of the double delete strain to AmB, CFW and CR (Fig. S2), confirming that the phenotypes of this strain are in fact due to deletion of these two genes. The double delete strain also showed very intense fluorescence after CFW staining (Fig. 3b); by semi-quantification, the fluorescence intensity of this strain was found to be 127% higher than that of the wild-type strain SN95. The *Cafen1*Δ/Δ and *Cafen12*Δ/Δ strains, respectively, showed 46% and 17% increase in fluorescence. These results indicate higher chitin content in the cell wall, particularly in the double delete strain. We also tested the sensitivity of these deletants to zymolyase, and as expected double deletant was more sensitive compared to single deletants and parent strain (Fig. 3c), and their relative sensitivity was comparable to their sensitivity to CFW (Fig. 3a), suggesting that chitin deposition is associated with defective synthesis or increased accessibility of β-1,3-glucan. Compactness of the cell wall was also assessed in these deletants by SDS sensitivity test. Double deletant was not growing at the tested concentrations of SDS, and similar to *Scsur4*Δ/Δ deletant, *Cafen12*Δ/Δ was more sensitive to SDS compared to *Cafen1*Δ/Δ and the parent strain (Fig. 3d). Altogether, these results reveal the functional importance of Fen1p and Sur4p in maintaining the CWI in both *S. cerevisiae* and *C. albicans*. Though double deletion of *FEN1* and *SUR4* orthologs is not lethal in *C. albicans*, unlike in *S. cerevisiae*^46^, synergistic sensitivity towards cell wall perturbing agents by deletion of *CaFEN1* and *CaFEN12* in *C. albicans* suggest that both these genes independently impair CWI. Individual deletion of *FEN1* and *SUR4* genes of *C. glabrata* and *S. cerevisiae* differentially affect susceptibility to echinocandins caspofungin and micafungin^47^. Moreover, the double delete (*Cafen1*Δ/Δ *Cafen12*Δ/Δ) strain of *C. albicans* also showed a similar phenotype^48^. Since echinocandins inhibit the 1,3-β glucan synthase activity, changes in the efficacy of these antifungals against these mutants further confirm the role of these genes in CWI.

### Compromised sphingolipid biosynthesis leads to cell wall defect

**Figure 4:**
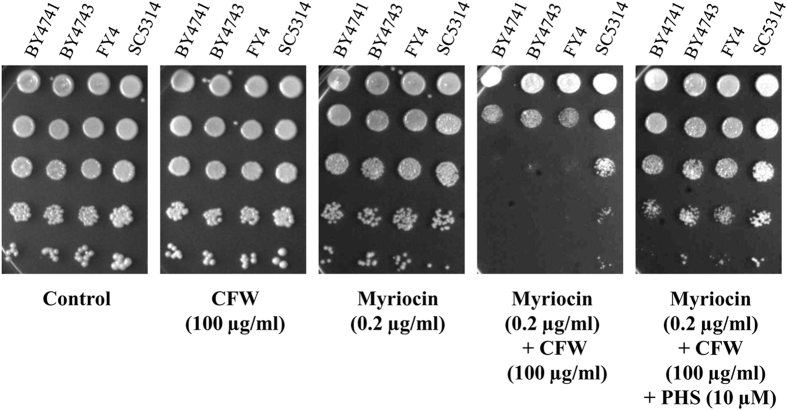
Sphingolipid depletion leads to cell wall defect. Wild-type strains of *S. cerevisiae* (BY4741, BY4743, FY4) and *C. albicans* (SC5314) were tested for their sensitivity to CFW alone or in the presence of myriocin or myriocin and phytosphingosine (PHS). The sublethal concentration of myriocin sensitised the cells to CFW, which is rescued by supplementation with PHS.

To gain further insight into the functional significance of sphingolipids for CWI, we checked the cell wall defect in the presence of myriocin, which is an inhibitor of serine palmitoyltransferase that catalyses the first committed step of sphingolipid biosynthesis^21^,^22^. Wild-type strains of *S. cerevisiae* (FY4, BY4743 and BY4741) and *C. albicans* (SC5314) were used to test myriocin effect on CWI. Myriocin, at sub-lethal concentration (0.2 μg/ml), rendered the cells sensitive to CFW (100 μg/ml), indicating cell wall defect (Fig. 4). To check if the cell wall defect was because of depletion of sphingolipids, we supplemented phytosphingosine (PHS; 10 μM) to combined myriocin and CFW treated cells. PHS is a downstream intermediate in sphingolipid pathway and is known to rescue the myriocin mediated inhibition of sphingolipid biosynthesis^49^. PHS reversed the myriocin mediated CFW sensitivity (Fig. 4). We have also tested the effect of myriocin and PHS on CFW sensitivity of delete strains (Fig. S3). While the deletants are as such more sensitive to CFW compared to wild-type strains (second panel), addition of myriocin further sensitised them to CFW (fourth panel). PHS seems to reverse only the additional sensitivity caused by myriocin (fifth panel), suggesting that it does not compensate for the lack of elongase activities of Fen1 and Sur4, though it suppresses depletion of sphingolipids and CFW sensitivity caused by myriocin (Fig. 4). These results confirm that the cell wall defect was indeed due to the depletion of sphingolipids.

### Cell wall defect is likely responsible for amphotericin B sensitivity of *FEN1* and *SUR4* deletants

**Figure 5:**
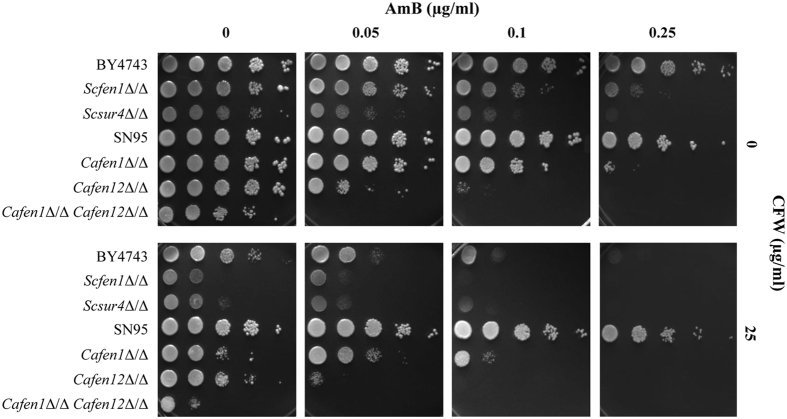
Cell wall perturbing agent calcofluor white sensitises cells to amphotericin B. Sensitivity of cells to AmB was checked without or with 25 μg/ml CFW.

We have previously reported that deletion of *FEN1* and *SUR4* genes leads to AmB sensitivity in *S. cerevisiae*, which was further validated in *C. albicans* deleted for their orthologs *CaFEN1* and *CaFEN12,* respectively^35^. Since alterations in the cell wall composition are known to affect AmB susceptibility^50^,^51^,^52^, it is likely that AmB sensitivity of these deletants is due to their cell wall defect. To check if directly modulating cell wall integrity would affect AmB sensitivity, we have tested the effect of CFW on AmB sensitivity. CFW was found to sensitise cells to AmB (Fig. 5), indicating cell wall damage can result in AmB sensitivity. To further assess the contribution of cell wall damage to AmB sensitivity, we tested deletants of three other genes that are not part of sphingolipid biosynthetic pathway but involved in CWI, for AmB sensitivity phenotype. *FKS1, GAS1* and *KRE6* are critical for cell wall biogenesis encoding β-1,3-glucan synthase, 1,3-beta-glucanosyltransferase, and type II integral membrane protein required for beta-1,6 glucan biosynthesis, respectively^53^. β-1,3-glucan synthase synthesises the β-1,3-glucan, which is further elongated and arranged in the side chains by 1,3-beta-glucanosyltransferase activity. *KRE6* encoded protein participates in the synthesis of β-1,6-glucan, which cross-links to the side chains of β-1,3-glucan and provides tight mesh structures of the cell wall. Deletion of these genes is known to be associated with cell wall defect and loss of CWI^53^, but their AmB sensitivity phenotype has not been reported so far. We tested AmB susceptibility of *fks1*Δ/Δ, *gas1*Δ/Δ and *kre6*Δ/Δ strains of *S. cerevisiae* and found that these were, in fact, sensitive to AmB (Fig. S4a). Normal transport of Gas1p and the other GPI-anchored proteins from ER to Golgi is reported to be abrogated in the deletants of *FEN1* and *SUR4* genes because of reduced sphingolipid biosynthesis^54^. Thus it appears likely that the AmB sensitivity of *FEN1* and *SUR4* mutants is due to weakened cell wall arising out of defective Gas1p transport. However, as these mutants would have shortened fatty acid chains and lower levels of sphingolipids^40^,^42^, we cannot rule out the contribution of these changes in the membrane lipid composition to the AmB sensitivity of cells. Overexpression of *PMP3* gene, encoding Plasma Membrane Proteolipid 3 protein, enhances AmB resistance^55^,^56^, which is also dependent on sphingolipid biosynthetic pathway^55^. Deletion of this gene renders the cells hypersensitive to AmB^55^,^56^, but unlike in *FEN1* or *SUR4* deletants, this does not involve any change in CWI^55^.

Several yeast genes such as PKC1, which are involved in CWI, when mutated render the cells osmotically fragile^19^. To check whether osmotic imbalance contributes to AmB sensitivity of *fks1*Δ/Δ, *gas1*Δ/Δ and *kre6*Δ/Δ strains, we tested them in the presence of sorbitol as osmotic support. Sorbitol failed to rescue the AmB sensitivity (Fig. S4a), implying that this phenotype is not due to osmotic imbalance. We also tested AmB sensitivity of *FEN1* and *SUR4* deletants of *S. cerevisiae* in the presence of sorbitol and found that their sensitivity was also not rescued by sorbitol (Fig. S4b). However, AmB sensitivity of *C. albicans* single gene deletants was partially suppressed by sorbitol (Fig. S4b), indicating that deletion of *CaFEN1* and *CaFEN12* leads to cell wall defect accompanied with osmotic destabilisation.

### *Candida albicans* strain deleted in both *CaFEN1* and *CaFEN12* is defective in hyphae and biofilm formation

**Figure 6:**
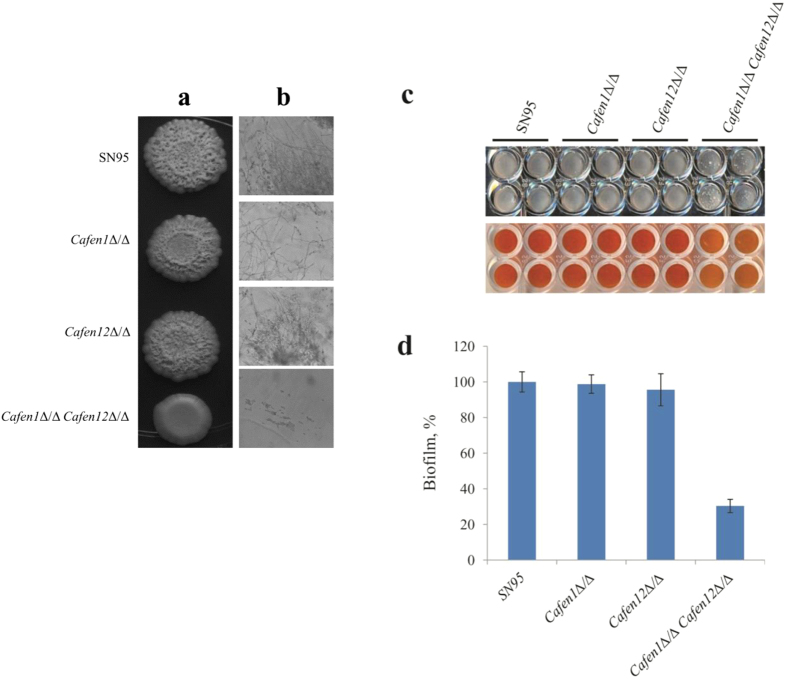
Double deletion of *CaFEN1* and *CaFEN12* genes impairs hyphae formation, invasive growth and biofilm formation in *C. albicans*. (**a**) Hyphae formation assay on YPD agar plate containing 10% fetal bovine serum. (**b**) Invasive growth into agar. After washing the cells on the agar surface with water, the agar was vertically sliced and observed under a microscope. (**c**) Biofilm formation. For each strain cells at a density of 1 × 10^6^ cells/ml were dispensed into 96-well microtiter plate in quadruplicate and incubated at 37 °C for 2 days. Then nonadherent cells were removed by washing, and leaving behind mature biofilm in the wells (upper panel). Metabolic activity of biofilms was visualised by conversion of XTT from light orange to dark orange colour (lower panel). (**d**) Quantification of biofilm formation by colorimetric XTT reduction assay. Average values of XTT reduction reading at 492 nm of each strain is expressed as a percentage of the value of parent strain. Error bars represent means ± standard deviations of three independent quadruplicate experiments.

**Figure 7:**
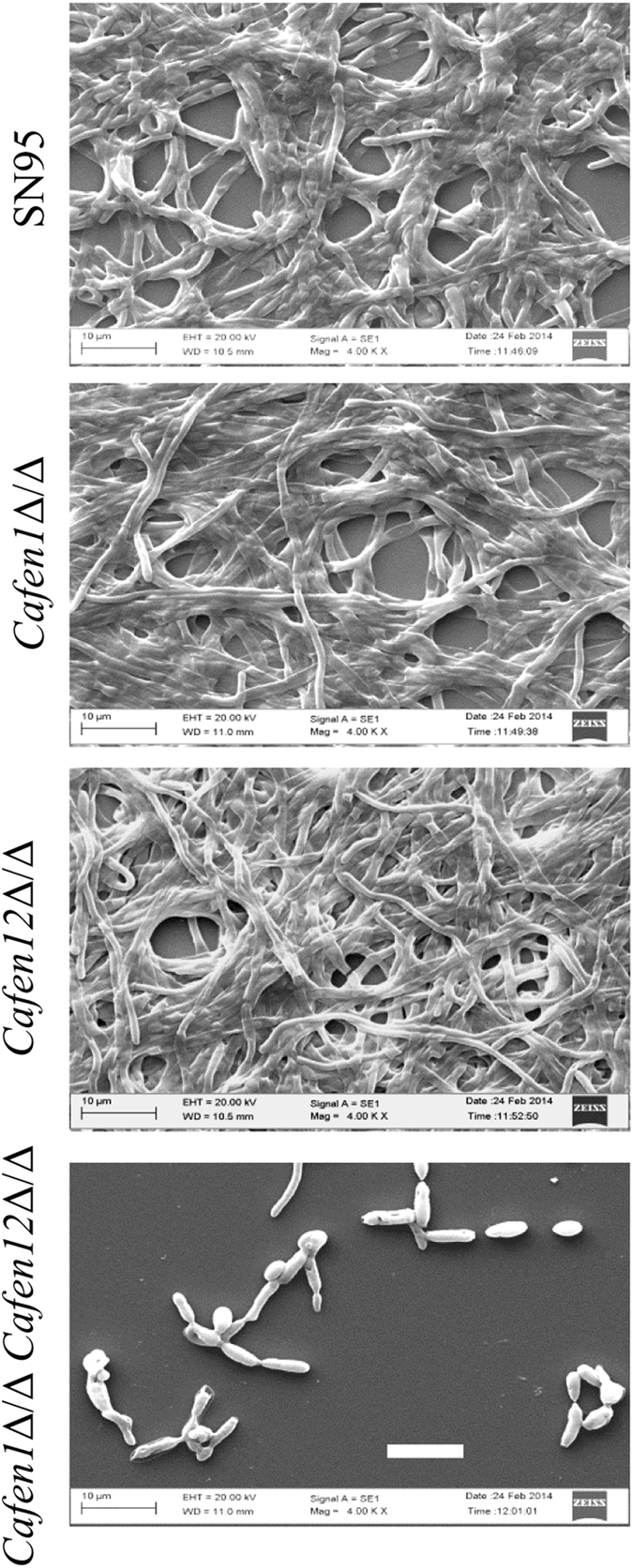
*CaFEN1*-*CaFEN12* double delete strain is defective in biofilm formation. Preformed biofilms of parent, single and double delete strains of *C. albicans* were visualised at 4000× magnification by SEM. Scale bar = 10 μm.

Deletion of both *FEN1* and *SUR4* genes in *S. cerevisiae* is lethal. However, both their orthologs could be deleted in *C. albicans* without loss of viability, but the strain was slow growing^35^. In a further phenotypic analysis of these strains, we tested their hyphal growth in solid medium (YPD agar) under hypha-inducing condition (10% FBS). While double deletant was deficient in hypha formation and invasive growth, the phenotypes of single deletants were comparable to the parent strain (Fig. 6a and b). Hyphal growth is a characteristic feature of biofilm development, and both are important for pathogenicity of *C. albicans*^57^,^58^,^59^. Biofilm is a complex three-dimensional structure consisting of yeast, pseudohyphae and hyphae, which are usually surrounded by a protective layer called extracellular matrix substance that adheres to the surfaces^57^,^60^. Immunocompromised patients with indwelling medical devices are more prone to *Candida* biofilm development^57^,^61^,^62^. Biofilm formation is considered to be one of the leading causes of candidiasis mediated mortality because of their natural antifungal resistance^57^,^61^,^62^,^63^. Since hyphae are integral to biofilm architecture, and hyphae formation is impaired in *Cafen1*Δ/Δ *Cafen12*Δ/Δ double deletant, we tested the effects of single as well as double deletion of *CaFEN1* and *CaFEN12* on biofilm formation in a microtiter plate with colorimetric XTT reduction assay. Biofilm formation was reduced by 70% in the double deletant as compared to the parent strain though no effect was seen for single deletants (Fig. 6c d and). Formed biofilms were also visualised by scanning electron micrography. While parent and single deletant strains showed typical biofilm architecture with extensive hyphae, double deletant strain lacked hyphal growth or biofilm formation but formed pseudohyphal like structure (Fig. 7). Inhibition of sphingolipid biosynthesis was reported to affect lipid rafts, hyphae and biofilm formation^64^. Thus, the defect in hyphal growth and biofilm formation in double deletant is likely due to impaired sphingolipid biosynthesis.

Previously^35^, as well as in this study, we showed that the planktonic cells of these deletants were more sensitive to the AmB as compared to the parent strain. Since biofilms are inherently more resistant to antifungals^57^,^61^,^62^,^63^, we tested AmB sensitivity of these deletants in preformed biofilms and during biofilm formation. We incubated preformed biofilms of the deletants and parent strain with AmB at 37 °C for 2 days and then determined the viability by XTT reduction assay. The AmB sensitivity of biofilms formed by single deletants was comparable to the parent strain. However, double deletant was 8-fold more sensitive to AmB compared to parent strain suggesting that the defect in biofilm formation rendered the cells more sensitive to AmB (Table 1). Moreover, we also tested the AmB sensitivity during biofilm formation, in which cell density comparable to that of biofilm formation was used. AmB was included from the beginning and incubation was done at 37 °C for 2 days to allow the formation of mature biofilms. At the end of incubation, non-adherent cells were aspirated and washed out, and remaining formed biofilms were quantified by XTT assay. During biofilm formation, single and double deletants were found to be 2-fold and 8-fold, respectively, more sensitive to AmB compared to the parent strain (Table 1). The enhanced sensitivity of double deletant appears to be due to its inability to form biofilms that are inherently more resistant to AmB^57^,^61^,^62^,^63^. Biofilm extracellular matrix constituents such as β-1,3-glucan and extracellular DNA are known contributing factors for AmB resistance^62^.

In conclusion, we have shown that *C. albicans* genes *CaFEN1* and *CaFEN12* involved in sphingolipid biosynthesis are critical for cell wall integrity and for the formation of hyphae and biofilm. The strain deleted in both these genes is highly sensitive to AmB, likely due to its weak cell wall and inability to form biofilm.

## Materials and Methods

### Yeast strains, media and growth conditions

Strains of *S. cerevisiae* and *C. albicans* used in this study are listed in Table 2. YPD, Synthetic complete (SC) and RPMI-1640 media were prepared and used as described previously^35^. For primary (overnight) or exponential culture strains were grown in YPD broth at 30 °C with agitation (200 rpm). Stock solutions of CFW (sigma), CR (sigma), AmB (Sigma), myriocin (Sigma) and PHS (TCI chemicals) were prepared in dimethyl sulfoxide (Sigma) and stored at −20 °C until use.

### Generation of *CaFEN1* and *CaFEN12* reintegration strains

Wild-type copy of *CaFEN1* was amplified from *C. albicans* SC5314 genomic DNA using primers CaFEN1-US1 (5′-CAATCATCGCACATAAAACC) and CaFEN1-DA2 (5′-GGTGATACATTTTTCGGAG). Similarly, wild-type copy of *CaFEN12* was amplified from the genomic DNA using primers CaFEN12-US1 (5′-ATAATGGAAGAGGGAAGGC) and CaFEN12-DA2 (5′- GTCATGTAGTTCCTGCTACC). Both the PCR products (0.5–1 μg) were separately transformed into the double delete strain SN95F1F12. The transformants were selected at 42 °C, a temperature at which the reintegrant strains would grow, but not the double delete strain^48^. The integration of *CaFEN1* and *CaFEN12* at their target loci was confirmed by diagnostic PCR using primers CaFEN1-DG-S (5′-CTCAATAGTCATCGACACG) and CaFEN1-DG-R1 (5′-GTGGTAGTCAAACCACTCCAC) for *CaFEN1* and primers CaFEN12-DG-S (5′-GAAGGATATGGAACATTCG) and CaFEN12-DG-R1 (5′-TCCATACTGCTCATGTTGAAG) for *CaFEN12*.

### Susceptibility testing by dilution spotting

Overnight grown yeast strains in YPD broth were re-inoculated in fresh YPD medium and incubated at 30 °C with shaking at 200 rpm. The exponential cells were harvested, washed with water and normalised to an optical density (OD_600nm_) of 1.0 (2 × 10^7^ cells/ml). These cell suspensions were ten-fold serially diluted, and 5 μl of each dilution was spotted on SC agar plates containing different concentrations of tested chemicals or drugs. Growth was assessed by incubating the plates at 30 °C for 2 days. Experiments were done at least thrice, with reproducible results.

### Fluorescence microscopy

Calcofluor white (CFW, Fluorescent Brightener 28, Sigma) was used as a fluorochrome having excitation and emission wavelength of λ_365_ and λ_435_ respectively. Exponential cultures of yeast strains were fixed with 4% paraformaldehyde for 30 min at 30 °C with agitation (200 rpm). The cells were then washed twice with sterile phosphate-buffered saline (PBS), resuspended in PBS with 10 μg/ml CFW and incubated for 15 min at room temperature in the dark. Following staining cells were washed twice with PBS and resuspended in the same buffer, before observing with a fluorescence microscope using 100× objective lens. NIS-Elements AR 3.2 software was used for semi-quantitative analysis of fluorescence intensity. Individual cells from different frames for a sample were encircled and the mean fluorescence intensity (MFI) of each cell was obtained using analysis controls. In the case of pseudohyphae formation, individual cells were selected instead of whole filaments. Average fluorescence intensity for each sample was calculated from MFI of all the cells of that sample.

### Zymolyase sensitivity assay

Zymolyase sensitivity of yeast strains was tested in pre-sterilized, polystyrene, flat-bottomed 96-well microtiter plates (Becton Dickinson), as described^65^, with some modifications. Yeast cells, equivalent to 0.5 OD_600nm_, were harvested from exponential culture, washed with PBS and resuspended in 200 μl zymolyase assay buffer containing 50 mM Tris-HCl (pH 7.5), 150 mM NaCl, 5 mM EDTA, 4% PEG (8000) and 2 U (100 μg) of zymolyase 20 T (Seikagaku Corporation, Japan)^65^. The microtiter plate was incubated at 30 °C with shaking in microtiter plate reader (BioTek Microplate Reader; USA) and OD_600nm_ was monitored for 90 min.

### Hyphae formation assay

It was performed on YPD agar plate containing 10% fetal bovine serum (FBS) (Invitrogen). Five μl of normalised 1.0 OD_600nm_ cells were spotted and incubated at 37 °C for 5 days before being photographed. To check invasive growth into agar, cells on the agar surface were washed away with water; the agar was then vertically sectioned and the agar slice was observed under a microscope with a 40× brightfield objective.

### Biofilm formation and XTT reduction assay

Biofilms were formed in a 96-well microtiter plate as described previously^66^,^67^. For inoculum preparation, exponential YPD broth cultures were harvested, and cells were resuspended in RPMI-1640 medium at a density of 1 × 10^6^ cells/ml. 100 μl of inoculums were dispensed into selected wells of 96-well microtiter plates and incubated at 37 °C for 2 days. After incubation, medium was gently aspirated from the wells and non-adherent cells were removed by washing thrice with sterile PBS. Residual PBS of wells was then removed by blotting with paper towels. Colorimetric XTT [2,3-bis(2-methoxy-4-nitro-5-sulfophenyl)-2H-tetrazolium- 5-carboxanilide sodium salt] reduction assay was then performed for the quantification of biofilm formation as previously reported^66^,^67^. Briefly, 1 μM final concentration of menadione (Sigma; 10 mM prepared in acetone) was added to the filter sterilised (0.22 μm filter) stock solution of XTT tetrazolium salt (0.5 g/L) (Sigma). 100 μl of XTT-menadione solution was added into the prewashed preformed biofilms and to empty wells (for the background values of XTT reduction) of microtiter plates and incubated at 37 °C in the dark for 1 hr. Colorimetric change in the XTT reduction was measured in a microtiter plate reader at 492 nm.

### Scanning electron microscopy (SEM)

For SEM, biofilms were formed on poly-L-lysine coated glass coverslips in 24-well cell culture plates (Nunc). 1 ml inoculums of 1 × 10^6^ cells/ml were dispensed into selected wells of a microtiter plate and incubated at 37 °C for 2 days. After incubation, biofilms were processed and dried as described previously^68^, with some modifications. Briefly, preformed biofilms were washed 3-times with PBS and fixed subsequently for 20 min with formaldehyde (4% vol/vol) and glutaraldehyde (2% vol/vol). After fixation, biofilms were dehydrated through a series of ethanol solutions (30%, 50% and 70% for 10 min, 20 min and 30 min respectively at 4 °C and with 90% and 95% ethanol for 30 min at room temperature). Final dehydration was carried out by t-butyl alcohol for 30 min at room temperature and then dried in a desiccator. The samples were then coated with gold-palladium for 135 sec at 10–12 milliamps and observed with a scanning electron microscope (ZEISS EVO 40) in high-vacuum mode at 20 kV.

### Amphotericin B susceptibility testing on preformed biofilms and during biofilm formation

For susceptibility assay on preformed biofilms, serially double-diluted AmB (0–16 μg/ml) in RPMI-1640 was dispensed (100 μl per well) into the wells of prewashed preformed biofilms and incubated at 37 °C for 2 days. For AmB susceptibility testing during biofilm formation, biofilms were formed in 96-well microtiter plates as described above with some modifications. For inoculums preparation, cells were resuspended in RPMI-1640 medium at a density of 2 × 10^6^ cells/ml. Inoculums were dispensed (100 μl per well) in serially double-diluted concentrations of AmB (0–16 μg/ml), such that final cell density is 1 × 10^6^ cells/ml for biofilm formation. At the end of incubation, AmB susceptibility of biofilm was measured by XTT reduction assay. AmB susceptibility experiments were performed on three different days in quadruplicates.

## Additional Information

**How to cite this article**: Alfatah, M. *et al*. Critical role for *CaFEN1* and *CaFEN12* of *Candida albicans* in cell wall integrity and biofilm formation. *Sci. Rep.* **7**, 40281; doi: 10.1038/srep40281 (2017).

**Publisher's note:** Springer Nature remains neutral with regard to jurisdictional claims in published maps and institutional affiliations.

## Supplementary Material

Supplementary Figures

## Figures and Tables

**Table 1:** Amphotericin B susceptibility test.

Strain	Biofilm-eradicating concentration (BEC^a^)	Biofilm inhibiting concentration (BIC^a^)
	AmB (μg/ml)	
SN95	8	0.50
*Cafen1*Δ/Δ	8	0.25
*Cafen12*Δ/Δ	8	0.25
*Cafen1*Δ/Δ *Cafen12*Δ/Δ	1	0.06

^a^BEC and ^a^BIC were defined as the lowest AmB concentration which causes ≥95% reduction in the metabolic activity of preformed biofilm (BEC) or during biofilm formation (BIC), compared to untreated control.

**Table 2:** Strains used in this study.

Strain	Description/genotype	Reference/Source
***S. cerevisiae***		
FY4	MAT a	69
BY4741	MAT a*; his3*Δ*1; leu2*Δ*0; met*15Δ*; ura3*Δ*0*	70Euroscarf
BY4743	MAT a/α*; his3*Δ*1/his3*Δ*1; leu2*Δ*0/leu2*Δ*0; lys2*Δ*0/LYS2; MET15/met15*Δ*0;ura3*Δ*0/ura3*Δ*0*	70Euroscarf
*fen1*Δ/Δ	BY4743; MAT a/α*; his3*Δ*1/his3*Δ*1; leu2*Δ*0/leu2*Δ*0; lys2*Δ*0/LYS2; MET15/met15*Δ*0;ura3*Δ*0/ura3*Δ*0; YCR034w::kanMX4/YCR034w::kanMX4*	36Euroscarf
*sur4*Δ/Δ	BY4743; MAT a/α*; his3*Δ*1/his3*Δ*1; leu2*Δ*0/leu2*Δ*0; lys2*Δ*0/LYS2; MET15/met15*Δ*0;ura3*Δ*0/ura3*Δ*0; YLR372w::kanMX4/YLR372w::kanMX4*	36Euroscarf
***C. albicans***		
SC5314	Wild-type clinical isolate	71
SN95	*arg4*∆*/arg4*∆ *his1*∆*/his1*∆ *URA3/ura3::imm*^*434*^*IRO1/iro1::imm*^*434*^	72
SN95F1	As SN95, *Cafen1*Δ::*lox/Cafen1*Δ::*lox*	35
SN95F12	As SN95, *Cafen12*Δ::*lox/Cafen12*Δ::*lox*	35
SN95F1F12	As SN95, *Cafen1*Δ::*lox/Cafen1*Δ::*lox Cafen12*Δ::*lox/Cafen12*Δ::*lox*	35
SN95F1F12-FEN1	As SN95, *Cafen1*Δ::*lox/CaFEN1 Cafen12*Δ::*lox/Cafen12*Δ::*lox*	This study
SN95F1F12-FEN12	As SN95, *Cafen1*Δ::*lox/Cafen1*Δ::*lox Cafen12*Δ::*lox/CaFEN12*	This study
