# Supplementary material for: Critical role for CaFEN1 and CaFEN12 of Candida albicans in cell wall integrity and biofilm formation
Source: Sci Rep. 2017 Jan 12;7:40281. doi: 10.1038/srep40281 (PMC5227966; doi:10.1038/srep40281)
Supplement: Supplementary Figures [file srep40281-s1.pdf]

Supplementary Figures

Critical role for *CaFEN1* and *CaFEN12* of *Candida albicans* in cell wall integrity and biofilm Formation

Md. Alfatah, Vinay K. Bari, Anubhav S. Nahar, Swati Bijlani and K. Ganesan

CSIR-Institute of Microbial Technology, Sector 39-A, Chandigarh-160 036, India

Contents:

Figure S1
Figure S2
Figure S3
Figure S4

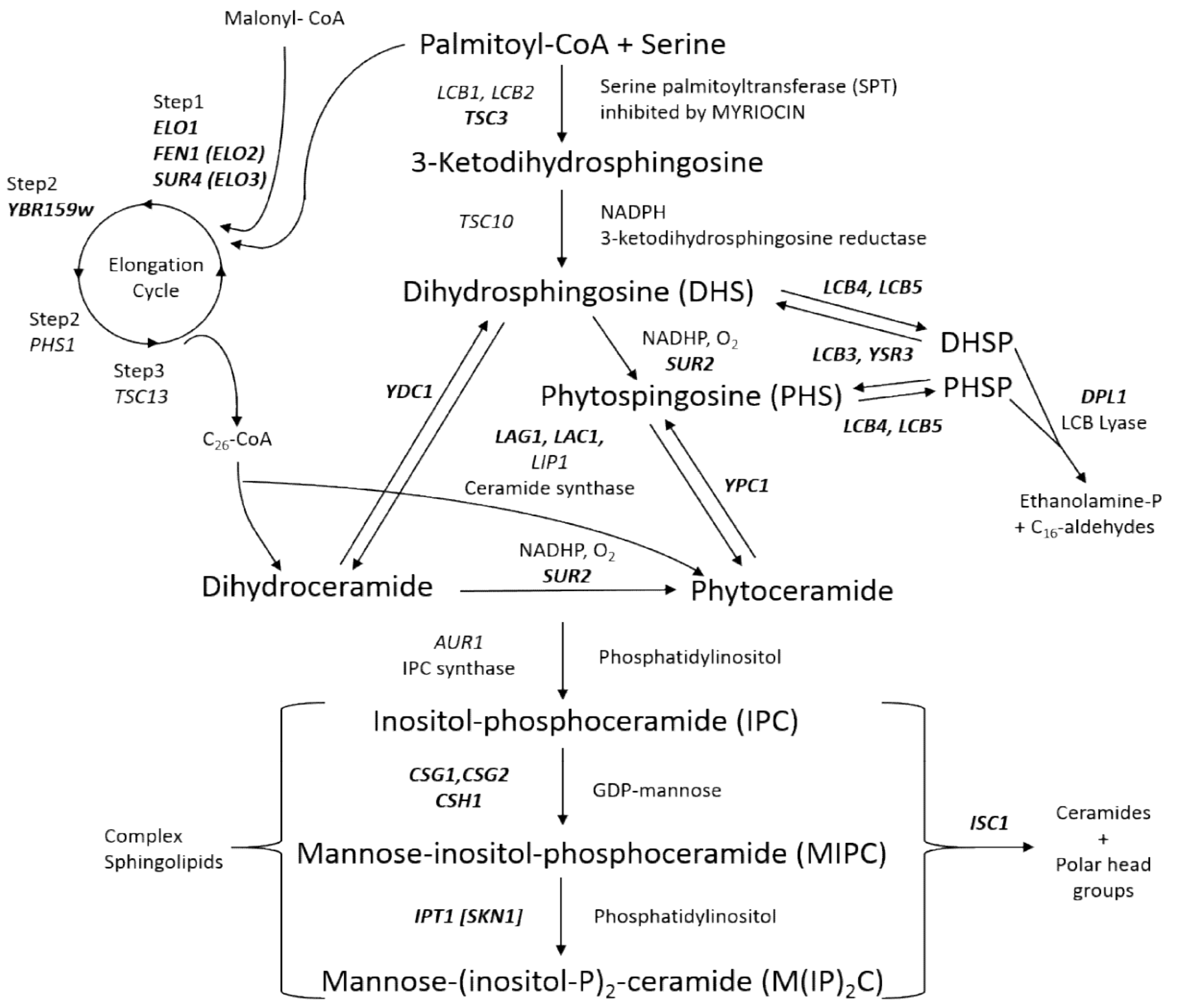

Figure S1. Overview of sphingolipid biosynthetic pathway of yeast. Genes that can be deleted without loss of cell viability are shown in bold font. *Adapted from:* Dickson, *Journal of lipid research* 49, 909-921 (2008), and Rego et al. *FEMS Yeast Res* 14, 160-78 (2014).

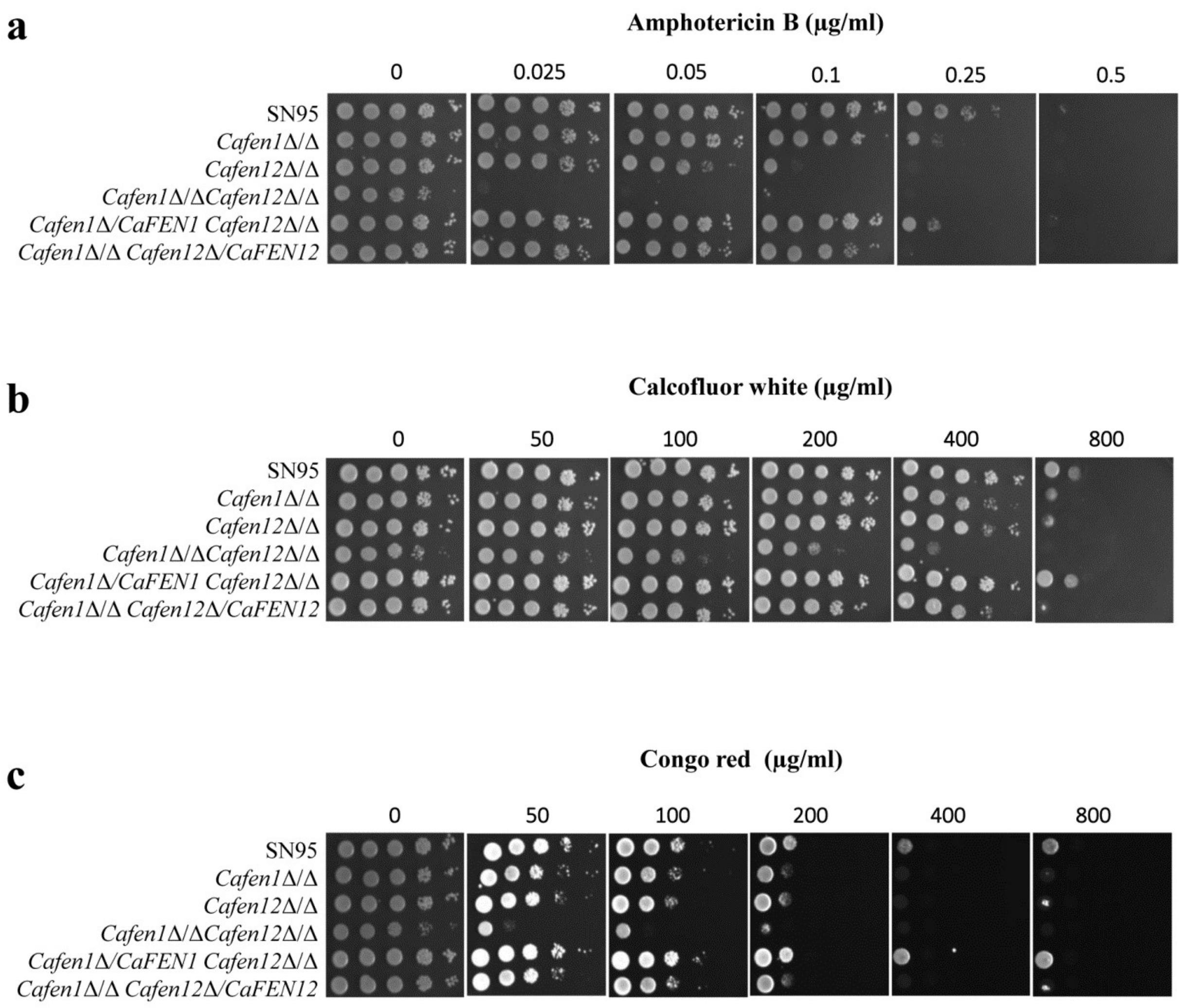

Figure S2. Sensitivity of double delete strain (*Cafen1*Δ/Δ*Cafen12*Δ/Δ) of *C. albicans* to amphotericin B (Panel **a**), calcofluor white (Panel **b**) and congo red (Panel **c**) is suppressed in the strains with reintegrated wild type copies of *CaFEN1* (*Cafen1*Δ*/CaFEN1 Cafen12*Δ/Δ) or *CaFEN12* (*Cafen1*Δ/Δ *Cafen12*Δ*/CaFEN12*).

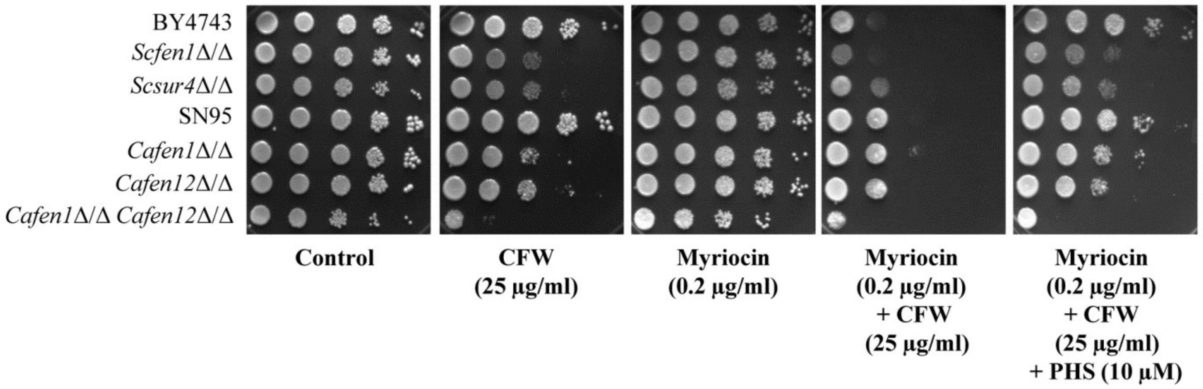

Figure S3. Sphingolipid depletion by myriocin sensitises cells calcofluor white.
S. cerevisiae strains deleted in *FEN1* or *SUR4* and *C. albicans* strains deleted in *FEN1* or *FEN12* or both were tested for their sensitivity to CFW alone or in the presence of myriocin or myriocin and phytosphingosine (PHS). The CFW sensitivity, enhanced by sublethal concentration of myriocin, is suppressed by PHS.

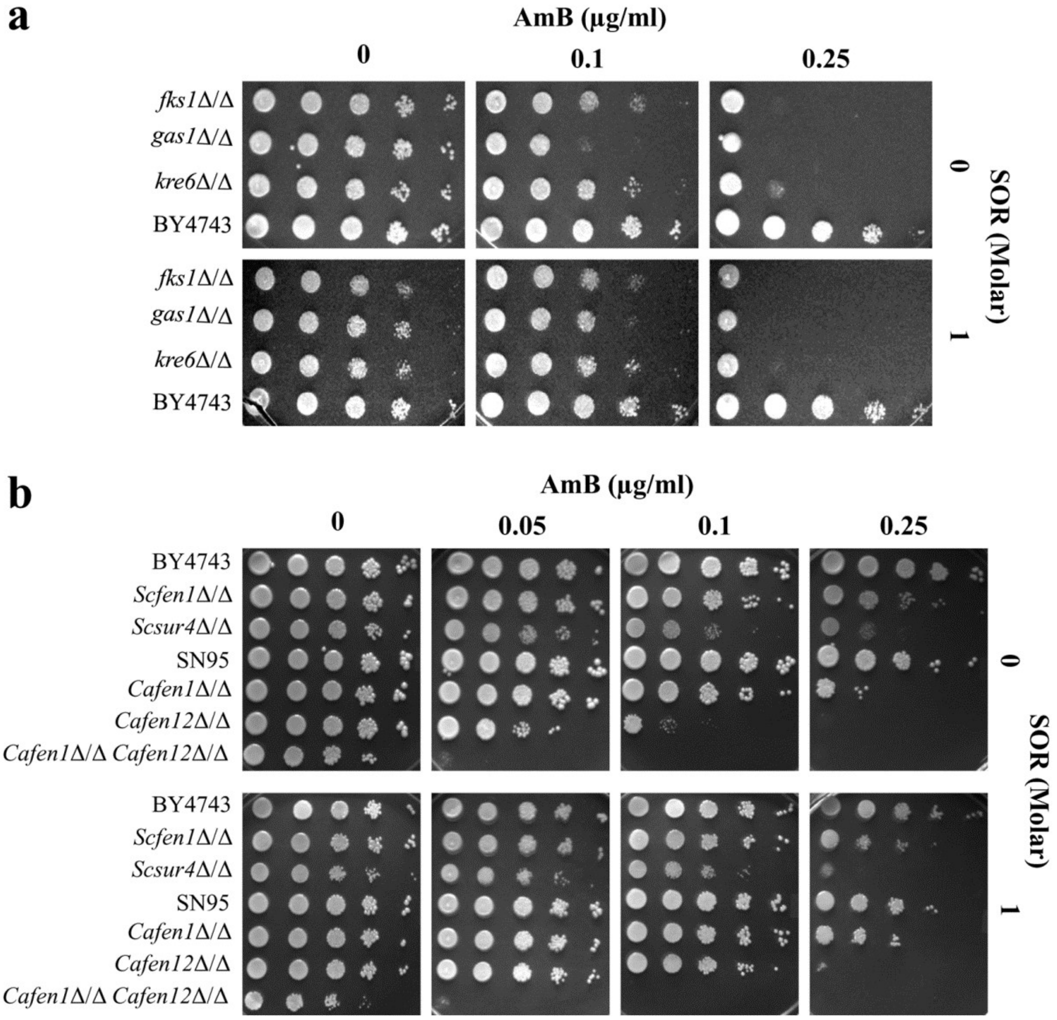

Figure S4. Effect of osmotic stabiliser on amphotericin B sensitivity. (**a**) AmB sensitivity of *S. cerevisiae* strains deleted in *FKS1*, *GAS1* or *KRE6* genes is not suppressed by sorbitol. AmB susceptibility of the parent (BY4743), *kre6*Δ/Δ, *gas1*Δ/Δ and *fks1*Δ/Δ strains were tested alone (upper panels) or in the presence of 1 M sorbitol (lower panels). (**b**) AmB sensitivity for *C. albicans* single strains deleted in *CaFEN1* or *CaFEN12* is partially reversed by sorbitol. Parent and delete strains of *S. cerevisiae* and *C. albicans* were tested for their sensitivity to AmB alone (upper panels) or in the presence of 1 M sorbitol (lower panels).
